# Avian Adeno-Associated Virus Vector Efficiently Transduces Neurons in the Embryonic and Post-Embryonic Chicken Brain

**DOI:** 10.1371/journal.pone.0048730

**Published:** 2012-11-07

**Authors:** Ryosuke Matsui, Yasuto Tanabe, Dai Watanabe

**Affiliations:** 1 Department of Molecular and Systems Biology, Graduate School of Biostudies, Kyoto University, Kyoto, Japan; 2 Department of Biological Sciences, Faculty of Medicine, Kyoto University, Kyoto, Japan; 3 Department of Developmental Neuroscience, Graduate School of Frontier Biosciences, Osaka University, Suita, Japan; McGill University, Canada

## Abstract

The domestic chicken is an attractive model system to explore the development and function of brain circuits. Electroporation-mediated and retrovirus (including lentivirus) vector-mediated gene transfer techniques have been widely used to introduce genetic material into chicken cells. However, it is still challenging to efficiently transduce chicken postmitotic neurons without harming the cells. To overcome this problem, we searched for a virus vector suitable for gene transfer into chicken neurons, and report here a novel recombinant virus vector derived from avian adeno-associated virus (A3V). A3V vector efficiently transduces neuronal cells, but not non-neuronal cells in the brain. A single A3V injection into a postembryonic chick brain allows gene expression selectively in neuronal cells within 24 hrs. Such rapid and neuron-specific gene transduction raises the possibility that A3V vector can be utilized for studies of memory formation in filial imprinting, which occurs during the early postnatal days. A3V injection into the neural tube near the ear vesicle at early embryonic stage resulted in persistent and robust gene expression until E20.5 in the auditory brainstem. We further devised an A3V-mediated tetracycline (Tet) dependent gene expression system as a tool for studying the auditory circuit, consisting of the nucleus magnocellularis (NM) and nucleus laminaris (NL), that primarily computes interaural time differences (ITDs). Using this Tet system, we can transduce NM neurons without affecting NL neurons. Thus, the A3V technology complements current gene transfer techniques in chicken studies and will contribute to better understanding of the functional organization of neural circuits.

## Introduction

The domestic chicken (*Gallus gallus domesticus*) has provided an attractive model system to understand the development and function of brain circuits. The chicks are born with their eyes open, and can actively explore from the moment they hatch [1]. This is because, as precocial birds, the precise organization of the functional brain circuits is mostly established during the embryonic period [2]. The chick embryo is useful, therefore, to study how precisely the development of complex brain circuits is programmed. In turn, postnatal chicks also offer a unique opportunity to explore the process of memory formation [3]. A visually naive chick immediately after hatching can easily learn the visual characteristics of a moving object, and subsequently form a strong social attachment to it [4]–[6]. Such imprinting memory has a great advantage in that the process of memory formation can be analyzed without interference from previous visual experiences [3]. To gain a further understanding of brain mechanisms using prenatal and postnatal chicks, development of sophisticated techniques for genetic modification of neuronal cells is important. Currently, electroporation-mediated and retrovirus (including lentivirus) vector-mediated gene transfer methods are widely used for chick studies [7]–[10]. These methods permit genetic manipulation with a relatively high efficiency during the early developmental stage [11], [12]. However, it is still challenging to efficiently and selectively deliver genetic material into chick postmitotic neurons without harming the cells.

In addition to the gene transfer techniques mentioned above, adeno-associated virus (AAV) vector is broadly applied to mammalian studies [13]–[15]. AAV is a naturally replication-defective, nonpathogenic single-stranded DNA virus [16] that can replicate only in the presence of a helper virus such as adenovirus or herpes virus [17]. The single stranded DNA of the AAV genome consists of two open reading frames (ORFs), *rep* and *cap* [18], and inverted terminal repeats (ITRs) at both ends of the DNA strand. The *rep* and *cap* ORFs encode four replication regulatory proteins and three capsid proteins, respectively [19]. The ITRs are the only cis-acting elements necessary for virus replication, packaging and integration [20]. Therefore, the recombinant AAV vector can be generated by transfecting host cells with a plasmid containing a transgene expression cassette flanked by the cis-acting ITRs and a plasmid expressing the *rep* and *cap* genes in trans, in the presence of helper virus genes [21].The recombinant AAV vector permits nontoxic transduction of postmitotic cells and long-term gene expression in neurons [22]. These properties, which make AAV vector one of the most attractive and promising vehicles for human gene therapy, also facilitate research of brain mechanisms. More than 100 variants of mammalian AAV have so far been identified [23]. Recombinant vectors made from these mammalian AAVs have been well characterized, and improved so as to achieve cell type-specific transgene expression [24]–[27] and to deliver genetically encoded tools for visualizing and manipulating neuronal activity [28], [29]. Unfortunately, these mammalian AAV vectors are not practical for chick studies, because transduction efficiencies of these mammalian AAVs are quite low in avian cells [30]. Recently, several strains of avian AAV (A3V) have been isolated, and their genomes were sequenced [30]–[32]. Because these A3Vs have a genome structure similar to that of the mammalian AAVs [30], [31], the basic strategy and process of generating recombinant vectors can be readily adapted from the mammalian counterparts [30], [31]. In principle, therefore, if a recombinant A3V vector could efficiently transduce chicken neurons, advanced technologies devised for mammalian AAV vectors could easily be introduced to chick studies. However, transduction characteristics of recombinant A3V vector in avian brain have been unexplored until now.

In this study, we demonstrate that the recombinant A3V vector efficiently transduces neuronal cells, but not non-neuronal cells in the chicken brain. We show that a single A3V injection into the postembryonic chick brain allows transgene expression selectively in neuronal cells within 24 hrs. Such rapid induction of gene expression raises the possibility that A3V vector can be utilized for studies of filial imprinting, which occurs during the early postnatal days [33]. We also found that A3V injection into the lumen of the neural tube near the ear vesicle can robustly transduce embryonic neurons in the auditory brainstem and the expression pattern of a transgene varies dependent on the embryonic stage of injection. Based upon these observations, we further developed an A3V-mediated tetracycline (Tet) dependent gene expression system [34] as a tool for studying the auditory circuit, consisting of the nucleus magnocellularis (NM) and nucleus laminaris (NL), that primarily computes interaural time differences (ITDs) [35]. Using this system, we established a method to selectively transduce NM neurons without affecting NL neurons. Neurons in the NM-NL circuit maintain a topographic arrangement [36], [37] similar to the organization of other sensory systems [38], and characteristic frequency-dependent differentiation and tuning of their cellular properties start during the embryonic stage [36], [39]. Thus, application of A3V-mediated gene transfer to chick embryonic auditory brainstem will also be useful to explore the development and information processing of the auditory system, a truly remarkable neural circuit that allows the precise computation of ITDs in the microsecond range.

## Results

### Efficient A3V Transduction of Postmitotic Neurons

**Figure 1:**
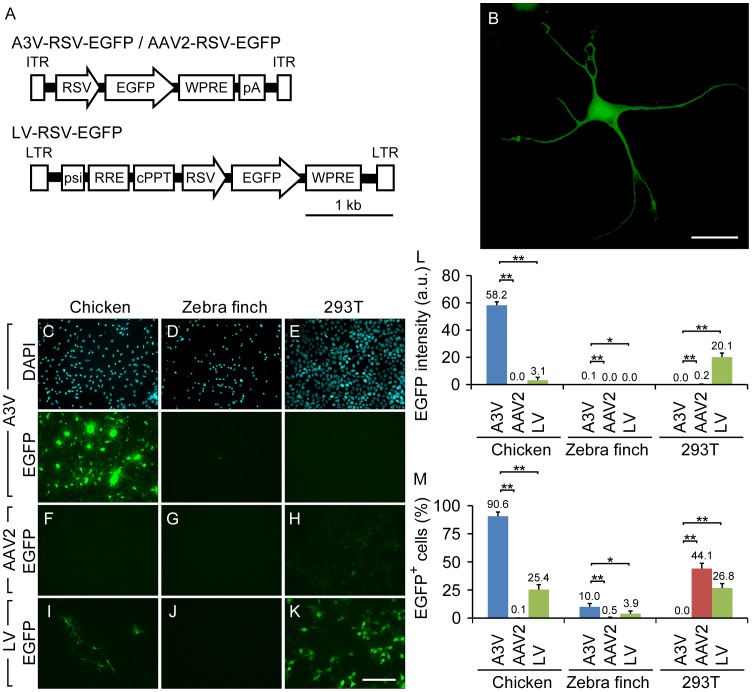
Comparison of transduction properties of A3V, AAV2 and LV. (A) Constructs of A3V-RSV-EGFP, AAV2-RSV-EGFP, and LV-RSV-EGFP. ITR, inverted terminal repeat; RSV, Rous sarcoma virus promotor; EGFP, enhanced green fluorescent protein; WPRE, woodchuck hepatitis virus post-transcriptional regulatory element; pA, SV40 polyadenylation signal; LTR, long terminal repeat; psi, packaging signal; RRE, Rev-responsive element; cPPT, central polypurine tract. (B) A representative example of EGFP-expressing cultured chicken neural cells after A3V treatment. Scale bar indicates 20 µm. (C–E) A3V-treated chicken neural cells, zebra finch neural cells, and 293T cells, respectively. Upper and lower panels represent the DAPI nuclear staining and EGFP fluorescent images of the same fields of view, respectively. All fluorescent images were taken with the same exposure time. (F–K) As a comparison, fluorescent images of corresponding cultured cells after AAV2 or LV treatment are shown. All images were taken with the same exposure condition as in (C–E). Scale bar indicates 100 µm. (L and M) Quantification of overall gene expression and transduction rate, respectively (n = 4). a.u., arbitrary units. *p<0.05; **p<0.005. The raw data are listed in Tables S1 and S2.

Previous studies have shown that recombinant A3V vector transfers genes into various types of chicken cell lines and primary cultured cells [30]. It was, however, unknown whether the A3V vector could transduce non-proliferative postmitotic neurons. To test its ability to transduce terminally differentiated neurons, we constructed an A3V vector that expresses EGFP under the control of the ubiquitous Rous sarcoma virus promotor (A3V-RSV-EGFP) (Figure 1A) and applied it to primary dissociated cultures from chicken embryonic brain. Postmitotic neurons, which are the majority of cultured brain cells and can easily be identified by their morphology, began to express EGFP as early as 24 hrs after the A3V-RSV-EGFP treatment, and EGFP fluorescence gradually increased up to at least 7 days (Figure 1B). Thus, the A3V vector can transduce gene expression in non-dividing chicken neurons.

To further evaluate the basic properties of the A3V vector, we compared its transduction with those of AAV2 and LV vectors, which are widely used to transduce various types of mammalian neural cells [40], [41]. We made AAV2 and VSV-G pseudotyped LV vectors that express EGFP under the control of the RSV promoter (AAV2-RSV-EGFP and LV-RSV-EGFP, respectively) (Figure 1A) similar to the A3V-RSV-EGFP. Each virus vector was applied to chicken neural cells, zebra finch neural cells and 293T cells, at a multiplicity of infection equivalent to 10^3^ genome copies (GC) per cell. Then, 3 days after infection, cells were fixed and counterstained with DAPI (upper panels in Figure 1C–E). Overall gene expression (average EGFP intensity) and transduction frequency (percent EGFP positive cells) were analyzed (Figure 1C–M). Quantification of average EGFP intensity (Figure 1L) and percent EGFP positive cells (EGFP^+^/DAPI^+^) (Figure 1M) revealed that A3V displayed the highest transduction efficiency in chicken and zebra finch neural cells among these three vectors, but A3V transduction in zebra finch cells is much lower than that in chicken cells (Figure 1L and M, and Tables S1 and S2). A3V had no apparent transduction activity in 293T cells (Figure 1L and M). On the other hand, AAV2 showed almost no transduction in the avian neural cells (Figure 1L and M). Because the only difference between the A3V-RSV-EGFP and AAV2-RSV-EGFP vectors is limited to the specific capsid proteins and the inverted terminal repeats (ITRs) flanking the gene expression cassette (RSV-EGFP), transduction efficiency and species-specificity of recombinant AAV vectors should be determined by the distinct structures of these limited components.

In contrast to the species-specific transduction by these AAV vectors, the LV vector transduced not only HEK293T cells but also an appreciable number of chicken neural cells (Figure 1I, K, L and M). However, the average EGFP intensity and transduction frequency in chicken neural cells were both much lower for LV than for A3V treatment (Figure 1L, and M). Furthermore, the morphology of EGFP-expressing primary cultured chicken cells appeared more variable in LV-transduced cells than A3V-transduced ones. Because primary brain culture contains non-neuronal cells such as astrocytes in addition to neurons, the differences in cell shape between the A3V- and LV-transduced cells may reflect their respective transduction selectivity to specific neural cell types.

**Figure 2:**
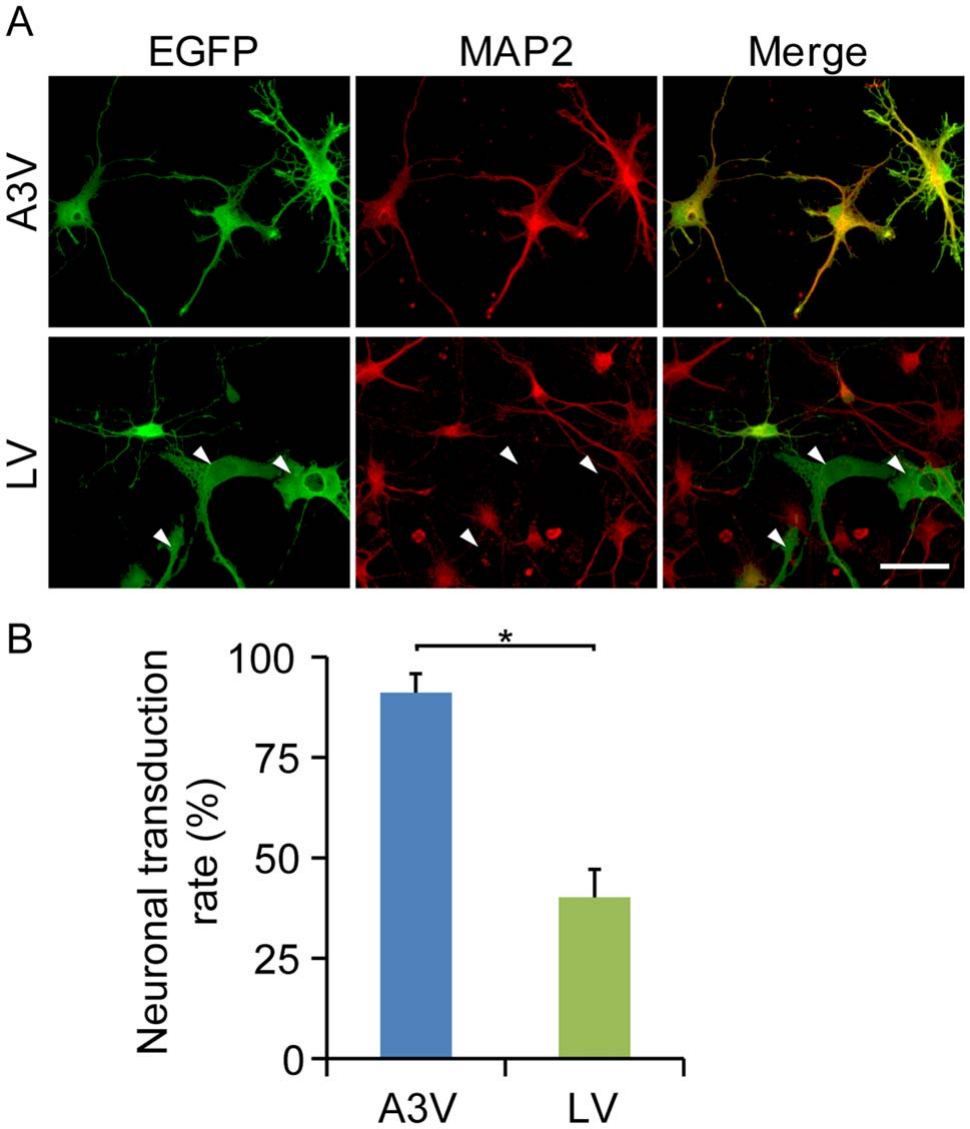
Neuron-preferential transduction of A3V vector. (A) Primary cultures of chick neural cells after A3V or LV infection were immuno-labeled with antibodies against EGFP (green) and the neuronal marker MAP2 (red). Arrowheads indicate MAP2-negative, LV-transduced cells. Scale bar indicates 20 µm. (B) The neuronal transduction rates of A3V and LV are represented as the percentage of MAP2 and EGFP double-positive cells within 100–200 EGFP-positive cells (n = 4). *p<0.005. The raw data are listed in Table S3.

As shown in Figures 1C and 1I, the intrinsic EGFP fluorescence was detectable after A3V or LV infection, but the intensity of the EGFP signal was quite different from cell to cell. To detect EGFP expression with high sensitivity and precisely assess transduction selectivity of the vectors for chicken neuronal and non-neuronal cells, we performed double immunofluorescence labeling against EGFP and neuronal marker MAP2, and determined the neuronal transduction efficiency (Figure 2A and B). EGFP-expressing cells after LV treatment included a considerable population (59.8%) of MAP2-negative non-neuronal cells (Figure 2B, Table S3). By contrast, more than 90% of EGFP-expressing cells in A3V-treated cultured cells were MAP2 positive (Figure 2B, Table S3), indicating that A3V efficiently transduces primary chick neurons in culture, but does not efficiently transduce non-neuronal brain cells. Because we used the ubiquitous RSV promoter, which is strongly active in a wide range of cells, the neuron-preferential transduction of the A3V vector is most likely due to the virus tropism of the A3V strain. Together, the A3V vector transduces postmitotic chicken neurons in a highly efficient and selective manner. These results point to its potential utility in chick studies *in* *vivo* and *in ovo*.

### A3V-mediated Gene Expression in Postnatal Chick Brain

**Figure 3:**
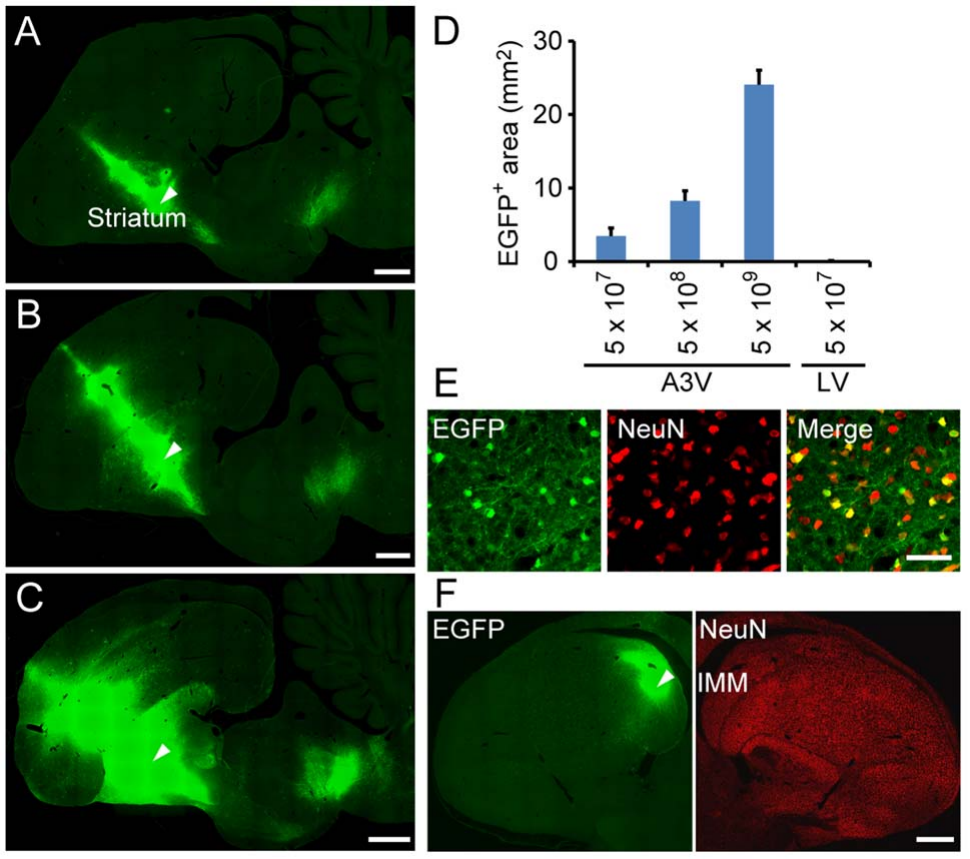
A3V gene transduction in post-hatch chick brain. (A–C) EGFP expression 1 week after A3V injection (a total of 5×10^7^, 5×10^8^, and 5×10^9^ GC, respectively) was analyzed by immunofluorescence labeling of parasagittal sections. Arrowheads indicate injection sites in the striatum. Scale bars are 1 mm. (D) Gene transduction after LV or A3V injection was quantified by measurements of EGFP-expressing area in the parasagittal sections containing injection sites (n = 4). The raw data are listed in Table S4. (E) A3V-treated striatal cells were visualized by double immunofluorescence labeling for EGFP (green) and neuronal marker NeuN (red). Scale bar indicates 50 µm. (F) EGFP expression 24 hrs after A3V injection (a total of 5×10^9^ GC) into the intermediate medial mesopallium (IMM) was analyzed by immunofluorescence labeling of coronal sections. The arrowhead indicates the A3V injection site. Scale bar is 1 mm.

Because young chicks can actively explore and rapidly learn from the moment they hatch, these precocial chicks have been considered as an attractive model to study neurobiological processes underlying memory formation [3], [4]. However, practical and efficient gene manipulation techniques in postnatal chick brain have not yet been established. To assess the utility of A3V vector, we characterized the transduction of A3V-mediated gene delivery to the chick brain after hatching. We injected 0.5 µl of a serially diluted A3V-RSV-EGFP viral stock (a total of 5×10^7^, 5×10^8^, and 5×10^9^ GC) into the unilateral striatum at post-hatch day 5 or 6 (PHD5 or 6), conducted immunofluorescence labeling of EGFP, and analyzed the effects of the transductions 7 days after injection. As shown in Figure 3A–C, administration of a fixed volume with increasing concentration of A3V particles to the striatum led to a commensurate increase in the EGFP expressing area. A strong EGFP signal was observed around the needle injection site after administration of 5×10^7^ GC (Figure 3A and 3D, Table S4), but was progressively more widely distributed throughout the forebrain after administration of 5×10^8^ and 5×10^9^ GC (Figure 3B, C, and 3D, Table S4). To examine neuronal transduction of A3V *in* *vivo*, we performed double immunofluorescence labeling for EGFP and the neuronal marker NeuN. As shown in Figure 3E, transduced cells around the injection site were almost 100% NeuN-positive (100.0±0.0% at 5×10^7^ GC, 98.2±0.8% at 5×10^8^ GC, and 100.0±0.0% at 5×10^9^ GC, n = 4). This indicates that the A3V vector can efficiently and selectively transduce chicken neurons not only *in* *vitro* but also *in* *vivo*.

Establishment of memory underlying filial imprinting is limited within the early postnatal days [33]. Therefore, rapid induction of gene expression is essential for the application of A3V-mediated gene transfer to studies of imprinting memory in these avian species. To address whether the A3V vector can allow gene expression within relatively short periods, we injected A3V-RSV-EGFP in the intermediate medial mesopallium (IMM), which is involved in memory for visual imprinting [42], at PHD0 and analyzed EGFP expression 24 hrs after injection. As shown in Figure 3F, EGFP fluorescence was observed in a considerable area (5.5±2.0 mm^2^, n = 4) within 24 hrs. To examine the transduction selectivity of A3V for neuronal cells in the IMM, we also performed immunofluorescence labeling for NeuN. All the EGFP-positive cells in the IMM expressed NeuN (100.0±0.0%, n = 4), indicating that A3V selectively transduces neurons in the IMM. These results demonstrate that A3V technology can become a powerful tool to study memory formation in filial imprinting that occurs during the early posthatch period.

### Gene Transduction in the Embryonic Auditory Circuit

**Figure 4:**
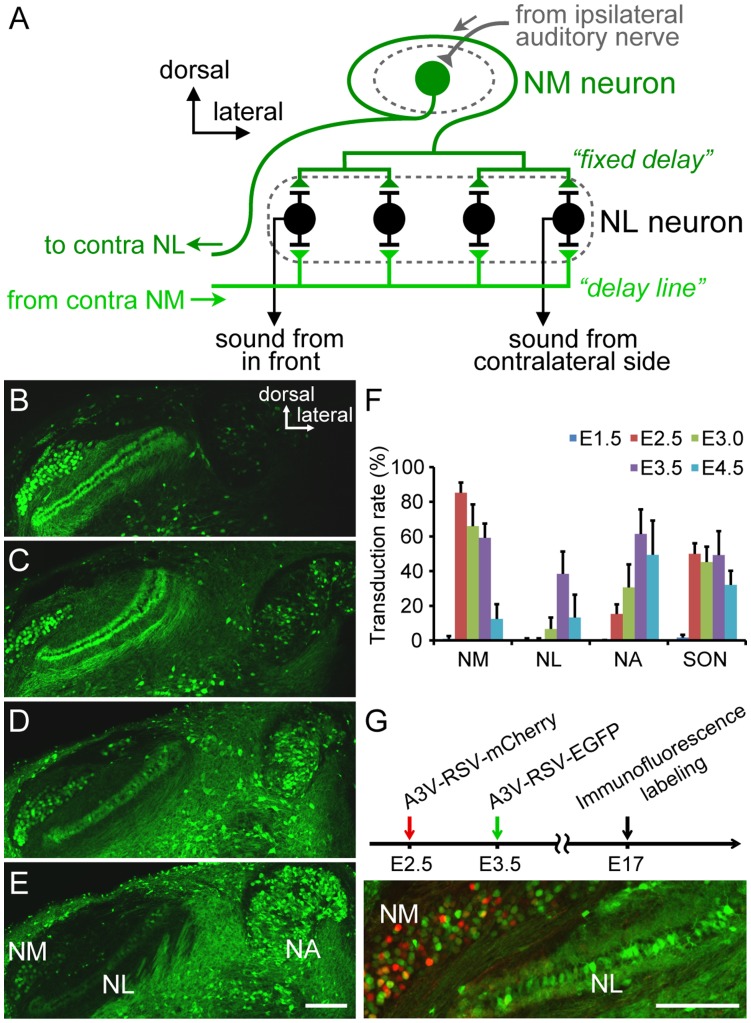
A3V-mediated gene transfer into the embryonic chick auditory brainstem. (A) Schematic of a primary ITD detection circuit composed of NM and NL neurons. The ipsilateral NM axons provide a simultaneous input to the dorsal side of the NL neurons, whereas the longer delay lines of contralateral NM axons project to the ventral side of more lateral NL neurons. Because NL neurons function as a coincident detector of binaural synaptic inputs, NL neurons in more lateral positions respond maximally to sounds originating in far contralateral space. (B–E) A3V-RSV-EGFP (0.5–1.5 µl, 1×10^12^ GC/ml) was injected into the neural tube at E2.5 (B), E3.0 (C), E3.5 (D), and E4.5(E), and EGFP signal at E17 was analyzed by immunofluorescence labeling of coronal sections. Scale bar indicates 200 µm. (F) A3V transduction rates in the embryonic auditory nuclei were quantified as the percentage of EGFP-expressing cells within total NeuN-positive cells in each nucleus (n = 6). The raw data are listed in Table S5. (G) A3V-RSV-mCherry and A3V-RSV-EGFP (0.5–1.5 µl, 1×10^12^ GC each) were injected at E2.5 and at E3.5, respectively. Spatial pattern of A3V transduction at E17 was analyzed by double immunofluorescence labeling for EGFP (green) and mCherry (red). Scale bars indicate 200 µm.

Animals utilize binaural interaural time differences (ITDs) in the microsecond range as a cue for localizing the sound source [35]. In avian species, NM and NL neurons in the brainstem form a primary circuit that computes ITDs (Figure 4A) [35], [43]. NM neurons receive monaural input from ipsilateral auditory nerve fibers and in turn project to bilateral NL neurons (Figure 4A) [36]. To calculate ITDs, NM axons provide the delay lines (Figure 4A) [37] and NL neurons precisely detect the coincidence of binaural synaptic inputs (Figure 4A) [35]. In addition, the NM-NL circuit is tonotopically organized [35] and the morphological and electrophysiological properties of both NM and NL neurons are specialized at each frequency [36], [39]. Previous studies have shown that such frequency dependent tuning in the ITD detection circuit has already started and has almost been established before hatching in the precocial birds [36], [39]. Thus, the chick embryonic auditory brainstem offers an elegant preparation in which to study mechanisms involved in the precise organization of functional brain circuits.

Because the embryonic central nervous system contains various types of neural cells in different differentiated states, transduction efficiency and neuronal selectivity of A3V may depend on the embryonic stage. We therefore examined how the transgene expression pattern in the embryonic auditory brainstem varies, dependent on the injection timing, and addressed whether A3V selectively transduces neurons in the developing auditory brainstem. A3V-RSV-EGFP was injected into the lumen of the neural tube near the ear vesicle at E1.5, E2.5, E3.0, E3.5, and E4.5, and EGFP expression in the auditory brainstem was assessed at E17. A3V injection at E1.5 scarcely induced EGFP expression in these auditory nuclei (data not shown), whereas injection at E2.5 to E4.5 resulted in persistent EGFP expression in the auditory brainstem nuclei until E17 (Figure 4B–E). To quantify transduction efficiency, we performed immunofluorescence labeling using anti-NeuN antibody and determined the frequency of EGFP-expressing NeuN positive cells (EGFP^+^ NeuN^+^/NeuN^+^) in the following auditory brainstem nuclei: NM, NL, nucleus angularis (NA), and superior olivary nucleus (SON). As summarized in Figure 4F (raw data in Table S5), the transduction efficiency of the individual auditory nuclei varied dependent on the injection timing. As a case in point, although NM and NL are very closely located in the early embryonic stage, these neighboring NM and NL showed quite different temporal profiles of transduction efficiency. NM neurons were transduced most efficiently at E2.5 (85.2±5.9%), and the transduction efficiency was considerably preserved untill E3.5 (59.2±8.2%). In contrast, EGFP expressing NL neurons were rare in the E2.5 injected samples (0.5±0.7%), and the maximum efficiency was obtained at E3.5 (38.4±12.9%). Such differences in temporal profiles of transduction rate may be because the individual NM neurons are sensitive to viral infection during a longer period than the NL neurons. We therefore examined whether successive A3V injection at different times can transduce the same NM neurons. We injected the A3V vector that expresses red fluorescent protein mCherry (A3V-RSV-mCherry) at E2.5 and then injected A3V-RSV-EGFP at E3.5 (Figure 4G, upper). As shown in Figure 4G, a considerable number of NM neurons expressed both mCherry and EGFP. Together, these observations suggest that the neighboring NM and NL may have quite different cellular properties in early on in embryonic stages. Next, to assess transduction selectivity of neuronal cells in the prenatal auditory brainstem, we analyzed the NeuN positive cell frequencies in the transduced cells (EGFP positive cells) in the NM, NL, NA, and SON. The NeuN positive cell frequencies (NeuN^+^ EGFP^+^/EGFP^+^) were almost 100% in these four nuclei (100.0±0.0% at NM; 99.2±1.9% at NL; 99.7±0.5% at NA; 97.6±3.0% at SON, n = 6), indicating that the A3V vector can transfer genetic material efficiently to neurons in the embryonic auditory brainstem.

### A3V-mediated Conditional Gene Manipulation

**Figure 5:**
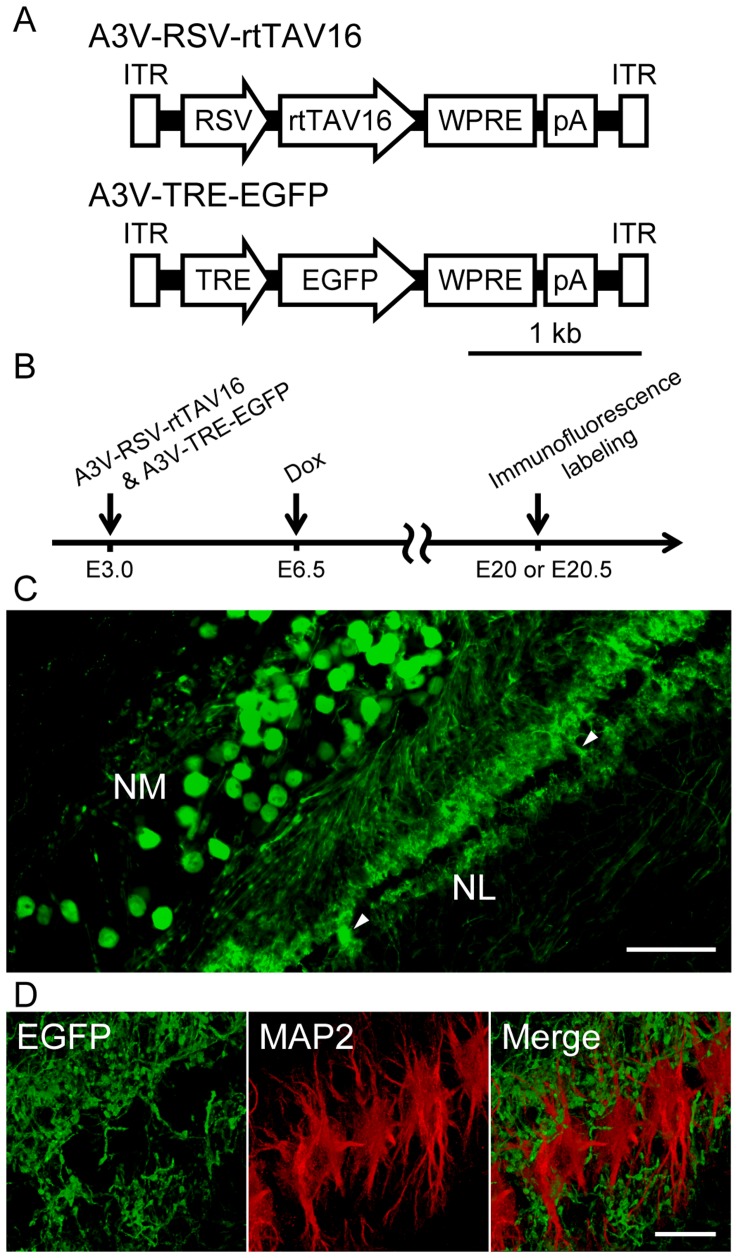
A3V-mediated Tet inducible expression system. (A) Tet inducible A3V constructs: rtTAV16, reverse tetracycline-controlled transactivator variant 16; TRE, tetracycline response element. (B) A3V-RSV-rtTAV16 and A3V-TRE-EGFP (0.5–1.5 µl, 1×10^13^ GC/ml each) were injected at E3.0, and Dox was administered at E6.5. Immunofluorescence labeling was conducted at E20 (n = 2 embryos) or E20.5 (n = 2 embryos). (C) Anti-EGFP immunofluorescence on a coronal section of the NL-NM circuits at E20.5. The EGFP signal was not detected in the cell bodies of the majority of NL neurons, but strong EGFP signal was observed in the cell bodies of some NL neurons (arrowheads). Scale bar indicates 100 µm. (D) A magnified view of NL neurons at E20.5, visualized with double immunofluorescence labeling for EGFP (green) and dendrite marker MAP2 (red). The immunofluorescence of EGFP was clearly separated from that of MAP2 in the NL dendritic portion. Scale bar indicates 20 µm.

Because NM axons serve as neural delay lines for ITD detection (Figure 4A) [37], the axonal conduction of NM neurons must be strictly regulated. To study the regulatory mechanisms and functional role of the axonal conduction of NM neurons, selective gene delivery to NM neurons without affecting the functional properties of NL neurons is useful. To achieve such selective expression of reporter genes, we exploited the difference in their sensitivity to A3V infection during the embryonic period (Figure 4F and G). We adopted a co-infection strategy to control gene expression in our A3V vector system. We constructed two A3V vectors comprising a tetracycline (Tet) dependent gene expression system (Figure 5A). One vector is A3V-RSV-rtTAV16, which expresses a recombinant transcriptional activator protein (rtTAV16) consisting of a modified Tet repressor [44] and a modified VP16 activation domain [34]. The other vector is A3V-TRE-EGFP, which expresses EGFP under the control of an inducible promoter composed of Tet response elements (TREs) and the cytomegalovirus (CMV) minimal promoter [45]. In this system, gene expression is activated only in the doubly infected cells as a result of Tet-dependent binding of the rtTAV16 protein to TREs located within the inducible promoter of A3V-TRE-EGFP.

First, to examine the sustained effect of a single injection of the Tet analogue doxycyclin (Dox), we performed A3V-TRE-EGFP and A3V-RSV-rtTAV16 injection into the neural tube at E3.0, applied Dox in the yolk at E6.0, and performed immunofluorescence labeling at E20 (n = 2 embryos) and E20.5 (n = 2 embryos) (Figure 5B). As shown in Figure 5C, a strong EGFP signal was observed in the NM-NL circuit at E20.5, indicating that gene induction after a single Dox injection can be sustained for more than 14 days. Furthermore, these results indicate that A3V injection at the early embryonic stage (E3.0) can lead to persistent gene expression through the late embryonic stage (E20.5) just before hatching. In the NL local circuit, only a few NL neurons expressed EGFP in their cell bodies (Figure 5C, arrowhead); EGFP expression was absent in the cell body portion of the majority of NL neurons, and enriched in their bipolar dendritic portion where the NM axons form synaptic contacts (Figure 5C). To examine the origin of the EGFP signal in the NL dendritic portion, double immunofluorescence labeling against EGFP and dendrite marker MAP2 was conducted. As shown in Figure 5D, EGFP immunofluorescence was clearly separated from MAP2 signal, indicating that the EGFP-labeled structures are NM axons, not NL dendrites.

**Figure 6:**
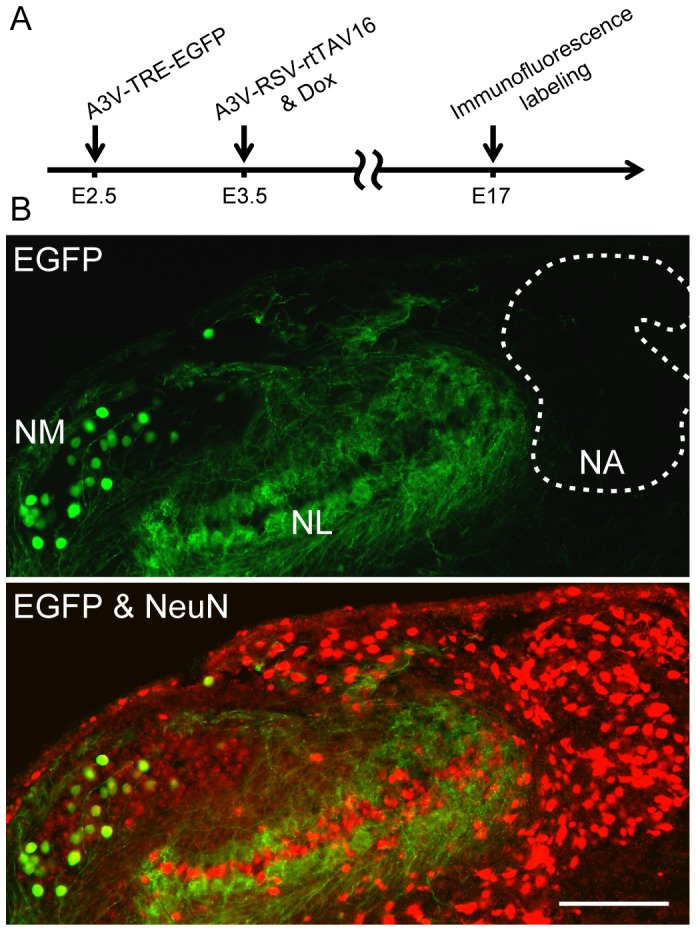
NM-selective transduction in the ITD-detection circuit. (A) Single doses of A3V-TRE-EGFP and A3V-RSV-rtTAV16 (0.5–1.5 µl, 1×10^13^ GC/ml each) were injected at E2.5 and at E3.5, respectively, and Dox was administered at E3.5. (B) Double immunofluorescence labeling for EGFP (green) and NeuN (red) was performed on coronal sections at E17. EGFP signal was observed in NM, but not in NL or NA. NA is outlined by the dotted line. Scale bar indicates 200 µm.

To more selectively transduce NM neurons in the NM-NL circuit, we next performed A3V-TRE-EGFP injection into the neural tube at E2.5, followed by A3V-RSV-rtTAV16 injection at E3.5, and then applied Dox in the yolk (Figure 6A). As shown in Figure 6B, EGFP-positive neurons were observed in NM, while none were observed in NL. In addition, EGFP expression was strongly suppressed in NA. Thus, the A3V-mediated Tet inducible expression system is useful to efficiently and selectively introduce genetic material into NM neurons without affecting NL neurons.

**Figure 7:**
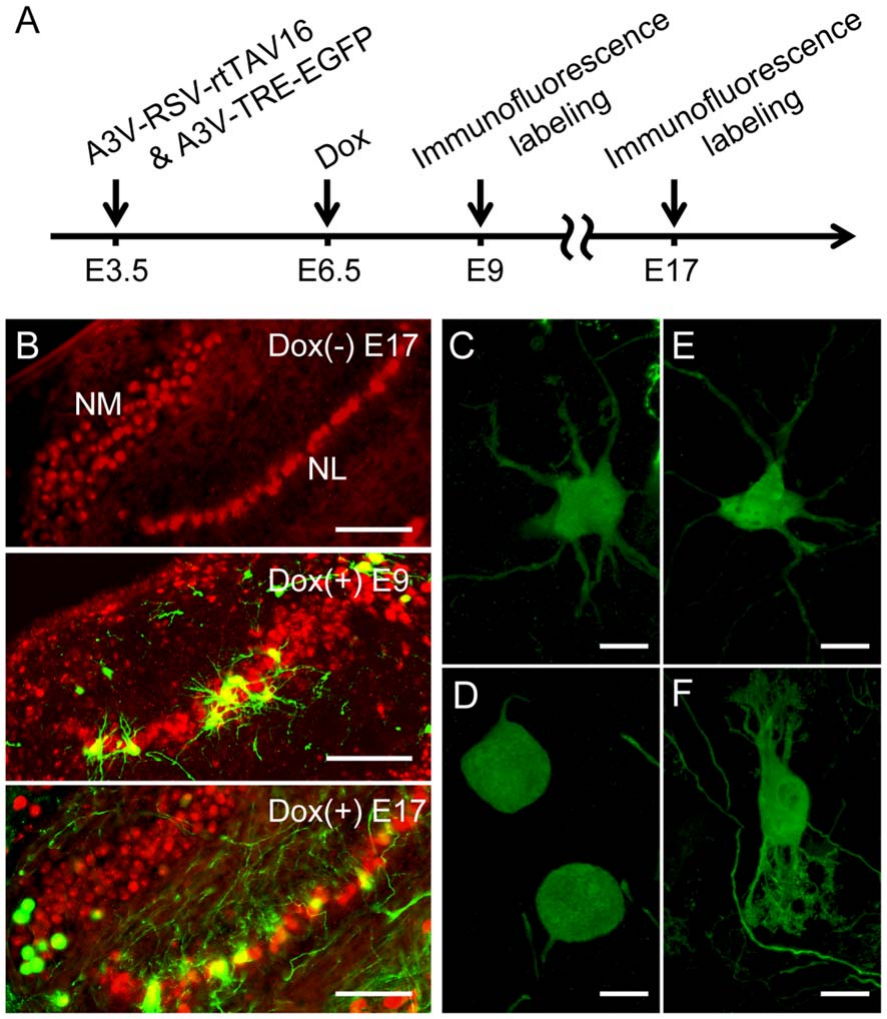
A3V-mediated Tet-inducible system robustly transduced a sparse population of NM and NL neurons. (A) A3V-TRE-EGFP and A3V-RSV-rtTAV16 (total 0.5–1.5 µl, 5×10^11^ GC/ml each) were injected at E3.5, and Dox was administered at E6.5. (B) Double immunofluorescence labeling for EGFP (green) and NeuN (red). No apparent EGFP signal was detected at E17 in the Dox (−) preparation (upper), while strong EGFP signal was observed both at E9 (middle) and E17 (bottom) in Dox (+) embryos. Scale bars indicate 100 µm. (C and D) Higher magnification views of EGFP-expressing NM neurons at E9 and E17, respectively. Scale bars indicate 20 µm. (E and F) NL neurons at E9 or E17, respectively. Scale bars indicate 20 µm.

During the embryonic stage, neurons in the NM and NL form tonotopically organized connections and show remarkable diversity in morphological properties depending on the characteristic frequency of sound. For morphological analysis of neural circuits, sparse and robust labeling of cells, such as Golgi staining, is useful. When diluted A3V-RSV-EGFP was injected, the EGFP signal in the auditory brainstem became sparse but weak (data not shown). To allow sparse and strong transgene expression in the NM and NL neurons, we employed the A3V-mediated Tet-inducible expression system. We injected A3V-TRE-EGFP and A3V-RSV-rtTAV16 at E3.5, followed by Dox administration at E6.5, and then EGFP expression was assessed at E9 and E17 (Figure 7A). As shown in Figure 7B (upper panel), without Dox administration, no apparent EGFP signal was detected at E17. In contrast, a single Dox treatment at E6.5 induced sparse and robust EGFP expression at E9, and the strong EGFP expression persisted until E17 (middle and lower panels in Figure 7B). As shown in Figures 7C–F, we could easily observe the morphological changes in the NM-NL circuit using EGFP; NM neurons showed extended ramifying processes at E9 (Figure 7C), while they lost their processes at E17 (Figure 7D); NL neurons were uniformly immature-appearing at E9 (Figure 7E), and displayed a characteristic bipolar shape at E17 (Figure 7F). Thus, the A3V-mediated Tet-inducible system permits us to introduce sparse and robust gene expression in developing NM and NL neurons in a temporally controlled fashion. Modifications allowing co-expression of an shRNA for gene knockdown or a mutant cDNA using a bicistronic construct could further elucidate the molecular mechanisms underlying tonotopic differentiation and organization of the ITD-detection circuit.

## Discussion

In this study, we characterized recombinant A3V vector-mediated gene transduction in chicken brain cells. In comparison with AAV2 and LV vectors, A3V showed highly efficient, selective transduction of differentiated neurons. A single injection of A3V vector into postembryonic chick brain induced gene expression selectively in neuronal cells within 24 hrs, suggesting that A3V can be applied as a gene delivery tool to study imprinting memory, which becomes established in a relatively short period of time after hatching. We also applied A3V technology to the primary neural circuit that computes binaural ITDs, and engineered a method to transduce NM neurons without affecting NL neurons during the formation of the ITD detection circuit.

A3V belongs to an AAV group (or *Dependovirus*) that is a naturally replication-defective DNA virus that can replicate only in the presence of a helper virus such as adenovirus or herpesvirus [17]. Because A3V has a genome structure that is common to all the other known mammalian AAVs, the basic strategy and process of generating recombinant vectors can be shared with mammalian AAVs [30], [31]. In fact, we could efficiently generate high-titer A3V vectors (up to 10^14^ GC/ml) based upon the standard protocols for mammalian AAV vectors (see materials and methods). Future advanced technologies devised for other AAV vectors could also easily be introduced to the design and production of the A3V vectors.

In contrast to the similarity in basic genome structure, transduction efficiency in avian neural cells was quite distinct between A3V and mammalian-derived AAV2 (Figure 1L and M). The only difference between A3V-RSV-EGFP and AAV2-RSV-EGFP is limited to their specific capsid proteins and ITRs, indicating that the different species-specificities between A3V and AAV2 are determined by the distinct structures of these two components. It may be possible that the ITRs of each respective virus strain could interact differently with intracellular mechanisms of the host cells. But as previously reported by Boissis et al., it is more likely that virus transduction is directly mediated by the capsid proteins that bind to cell surface molecules on the host cells [30]. The presumed exterior surfaces of AAV capsids have several divergent regions [30], and these divergent regions of A3V may exclusively recognize avian-specific cell surface molecules. The molecular mechanisms of AAV infection are not fully understood, but previous studies suggest that several different cell surface molecules are thought to collaboratively function as receptors for AAV infection [46]–[48]. The difference in A3V neural transduction between chickens and zebra finches may be determined by the difference in receptor molecules between these two species.

In addition to efficient transduction of avian cells in culture, the A3V vector showed neuron-specific transduction in the chicken brain (Figure 2 and 3E). Cell type specificity of gene expression is determined by the promoter and/or viral tropism. Because we used the ubiquitous RSV promoter that is strongly active in a wide range of cell types [49], the neuron-selective transduction of the A3V vector is probably due to the tropism of this A3V strain. In this study, we have demonstrated that A3V can transduce neurons in various brain structures, including striatum (Figure 3A–C), cerebral cortex (Figure 3F), and brainstem (Figure 4B–E). However, we did not determine whether A3V can infect all neuronal cell types. A previous report demonstrated that mammalian AAV vectors display subtype-specific tropism even within a restricted brain structure [50]. Therefore, A3V may also have such subtype specificity in neuronal transduction. A full understanding of the A3V tropism will require further studies focused on the diversity of cell types within each local circuit.

During the embryonic period, the developing nervous system contains undifferentiated neural cells along with postmitotic neurons. A3V vector injection at E2,5 to E4.5, however, resulted in neuron-specific transduction in the auditory brainstem. Such neuronal selectivity in the embryonic stage may be different from neuronal tropism observed in cultural neural cells or post-natal brain. Because A3V is a non-integrating virus [51], neuron-selective expression during the embryonic period may be partially due to a dilution effect on viral DNA copy number in actively proliferating undifferentiated neural cells. We also found that the gene expression pattern in auditory brainstem was dramatically altered dependent on the embryonic stages of A3V injection (Figure 4B–E). Although NM and NL are spatially very close to each other in the early embryonic stage, these neighboring NM and NL displayed quite different temporal profiles of A3V transduction efficiency (Figure 4F), indicating that NM and NL neurons have distinct properties in the early embryonic stage [52]. Based upon this observation, we successfully developed a method to introduce genetic material into NM neurons without affecting neighboring NL neurons, using our A3V-mediated Tet-inducible expression system. The fundamental role of the binaural auditory system is to localize sound sources by comparing the differences between the sound waves arriving at the two ears. NL is the first site that receives bilateral auditory information through the NM [35], and the NM-NL circuit functions as the primary detector of ITDs [43]. Because the axons of NM neurons function as the neural delay [37], axonal conduction of NM neurons should precisely be regulated to allow NL neurons to detect coincidently bilateral synaptic inputs. A three-dimensional reconstruction of the chick NM fibers suggests that both axonal diameters and internodal distances, as well as the axonal length, play a fundamental role in creating the proper neural delay [53]. A3V-mediated gene manipulation of NM neurons without affecting NL neurons (Figure 6) will be a powerful tool to study the delay tuning mechanisms for ITD-detection. We also applied this A3V-mediated Tet-inducible system to robustly transduce a sparse population of NM and NL neurons in a temporally controlled fashion (Figure 7). In addition to fluorescent reporter genes such as EGFP, we may co-introduce shRNA for gene knockdowns using a bicistronic Tet-inducible construct [54]. This would be helpful to further determine the molecular mechanisms underlying tonotopic differentiation and organization of the ITD-detection circuit.

In this study, we describe a novel virus vector system that efficiently delivers genetic material into prenatal and postnatal chicken neurons. The A3V technology will complement current gene transfer techniques in chick studies and will contribute to a better understanding of functional organization of neural circuits during and after the embryonic stages. In addition to overexpression or silencing of a gene of interest, A3V-based vectors can introduce genetically encoded tools into neurons for visualizing and manipulating activity, which has already been achieved with mammalian AAV vectors [55]–[58]. This will provide an excellent platform to address important issues in neuroscience.

### **Materials and Methods**

All procedures were in accordance with the National Institutes of Health *Guide for the Care and Use of Laboratory Animals* and were approved by the Institutional Animal Care and Use Committee of Kyoto University.

### Cell Culture

Human embryonic kidney 293T cells (RIKEN BioResource Center, Tsukuba, Japan, Cell No. RCB2202) were maintained at 37°C with 5% CO_2_ in Dulbecco’s modified Eagle’s medium (DMEM) supplemented with 10% fetal bovine serum (FBS; Biowest, Paris, France), 100 units/ml penicillin, and 100 µg/ml streptomycin.

Primary cultures of chicken and zebra finch brains were prepared essentially by the methods described earlier [59], [60]. The forebrain from embryonic day 7.5 (E7.5) chick or post-hatch day 0–1 (PHD0-1) zebra finch were dissected out and incubated in PBS containing 30 U/ml papain (Nakarai tesque, Kyoto, Japan) and 57 U/ml DNase I (Sigma-Aldrich, St. Luis, MO, USA) for 30 minutes (min) at 37°C. After rinsing three times in PBS, tissues were gently triturated with a 5 ml pipette 15 times, followed by a 1,200 µl filtered long tip 15 times. Then dissociated cells were passed through a cell strainer with a 40 µm nylon mesh (BD Falcon, Bedford, MA, USA), suspended with Neurobasal medium (Life Technologies, Grand Island, NY, USA) supplemented with 0.5 mM L-glutamine (Wako Pure Chemicals, Kyoto, Japan), 2% B-27 supplement (Life Technologies), 100 units/ml penicillin, and 100 µg/ml streptomycin. Cells were plated on 24-well plates coated with poly-L-lysine (10^5^ cells/well). The medium was refreshed 1 hr after plating to remove excess debris. Half of the medium was changed after 3 days in vitro (DIV3).

### Plasmid Constructs

AAV transfer vector plasmids (pA3V-RSV-EGFP, pA3V-RSV-mCherry, pA3V-RSV-rtTAV16) were constructed by replacing the β-galactosidase gene of pA3V-RSV-β-Gal [30] (kindly provided by Dr. J. Chiorini, NIH, Bethesda, USA) with the EGFP cDNA of pEGFP-N1 (Clontech, Mountain View, CA, USA), the mCherry cDNA of pmCherry-N1 [61] (kindly provided by Dr. R. Tsien, University of California, San Diego, CA, USA), and the rtTAV16 (described below), respectively. The AAV transfer vector plasmid pA3V-TRE-EGFP was constructed by replacing the RSV promotor sequence of pA3V-RSV-EGFP with the TRE promoter of pTRE-Tight (Clontech). The rtTAV16 was generated by introducing V9I, G12S, F67S, F86Y, R171K, and A209T mutations into the rtTA2^S^-M2 gene of pTet-On advanced vector (Clontech) so as to increase the sensitivity for doxycycline (Dox), as previously reported [34]. pAAV2-RSV-EGFP was constructed by inserting the fragment containing the RSV promoter and the EGFP cDNA of pA3V-RSV-EGFP into the multi-cloning site in pAAV-MCS (Agilent Technologies, Santa Clare, CA, USA). The woodchuck hepatitis virus post-transcriptional regulatory element (WPRE) of the pFUGW (kindly provided by Dr. D. Baltimore, California Institute of Technology, Pasadena, CA, USA) was then inserted before the SV40 polyadenylation signal (SV40 pA) in these AAV transfer vector plasmids. pLV-RSV-EGFP was constructed by replacing the ubiquitin promoter and GFP cDNA of pFUGW with the RSV promoter and the EGFP cDNA of the pA3V-RSV-EGFP.

### Generation of Recombinant Viral Particles

For the production of AAV2 and A3V, 293T cells were plated at 6×10^6^ cells per 15 cm dish the day before transfection. The medium was replaced with fresh medium 1–2 hrs before transfection. 293T cells were co-transfected with transfer vector plasmid, rep/cap-expressing plasmid and helper plasmid (rep/cap-expressing plasmid for A3V, provided by Dr. J. Chiorini; for AAV2, purchased from Agilent Technologies) by calcium phosphate precipitation (100 µg each for 12 dishes). The medium was replaced with DMEM containing 2% FBS 16–20 hrs after transfection. Cells were harvested 48 hrs after transfection, and pelleted by centrifugation at 1,100×g for 5 min. The pellets were lysed in 2.5 ml of 150 mM NaCl, 100 mM Tris-HCl pH 8.0 by a triple freeze-and-thaw procedure. The cell lysate was treated with 250 U/ml of benzonase (Merck, Darmstadt, Germany) for 30 min at 37°C and centrifuged at 7,900×g for 60 min. The virus-containing supernatant was further purified by iodixanol (OptiPrep; Axis-Shield, Oslo, Norway) step gradient ultracentrifugation [62]. After ultracentrifugation in a Beckman SW41 rotor at 40,000 rpm for 3 hrs, about 1.5 ml of the 40% iodixanol step was collected. Then the buffer was exchanged by dialyzing three times against PBS containing 0.001% Pluronic F-68 (Sigma-Aldrich) and the solution was concentrated down to 120 µl using a Vivaspin 20 (100,000 MWCO, Sartorius Stedim Biotech, Aubagne, France). The viral solution was clarified by centrifugation at 18,500×g for 5 min and stored at 4°C until used. Because there are no appropriate cell lines for measuring functional titers of both the A3V and AAV2 vectors, we did not determine their actual infectious titers, which are usually lower than genome titers by a factor of 10 to 1000 [30], [50], [63]. The DNase-resistant viral genome titers [64] were determined by quantitative real-time PCR (qPCR) using the following primers and a probe specific for the WPRE sequence: forward primer, 5′-CCGTTGTCAGGCAACGTG-3′; reverse primer, 5′-AGCTGACAGGTGGTGGCAAT-3′; probe, 5′-FAM-TGCTGACGCAACCCCCACTGGT-TAMRA-3′ [65], [66]. The yield of A3V and AAV2 was approximately 10^13^–10^14^ genome copies (GC)/ml.

VSV-G-pseudotyped lentivirus particles were produced basically as previously described [67], [68]. 293T cells were co-transfected with 133 µg of pLenti-RSV-EGFP-WPRE, 87 µg of pMDL, 47 µg of pVSV-G and 33 µg of pREV (provided by Dr. D. Baltimore) for 12×15 cm dishes by the calcium phosphate precipitation method. The medium was replaced with DMEM containing 2% FBS and 10 mM sodium butyrate (Wako Pure Chemicals) 16–20 hrs after transfection. Forty-eight hrs after transfection, the culture supernatant was harvested, clarified by centrifugation at 4,200×g for 5 min and filtered through a 0.45-µm filter (Corning, NY, USA). Then the sample was concentrated using a Vivaspin 20 (100,000 MWCO) and pelleted by ultracentrifugation in a Beckman SW41 rotor at 25,000 rpm for 2 hrs through a 20% sucrose cushion. The viral pellet was resuspended in 120 µl of PBS and stored in aliquots at −80°C until used. The viral RNA titers were determined by one-step qPCR with the primer/probe set for the WPRE sequence. The range of RNA titers was 10^12^–10^13^ GC/ml.

### Analysis of Transgene Expression in vitro

A single dose of 10^8^ genome copies (GC) of each virus vector was added to cultured neuronal cells at DIV3, or 293T cells soon after plating on 24-well plates at a density of 10^5^ cells/well. For the quantitative analysis of virus transduction, cells 3 days after viral infection were fixed with 4% paraformaldehyde in 0.1 M phosphate buffer (pH 7.4) for 30 min. To measure average EGFP intensity, fluorescent images of 3 random fields per well were taken with the same exposure using a 10×objective and a BZ-9000 fluorescence microscope (Keyence, Osaka, Japan). The background fluorescence was subtracted from each image using NIH ImageJ software (National Institute of Health, Bethesda, MD, USA) [69] and average fluorescence intensity per pixel was calculated. To examine transduction efficiency of neuronal transduction, we performed immunofluorescence labeling. Cells were permeabilized and blocked with PBS containing 10% normal goat serum and 0.1% Triton X-100 at room temperature for 60 min, and washed once with PBS. Cells were then incubated with primary antibody at 4°C overnight, and subsequently with secondary antibody at room temperature for 60 min. After washing three times in PBS, cells were counterstained with DAPI (4′,6-Diamidino-2-phenylindole; Dojindo Laboratories, Kumamoto, Japan) to identify cell nuclei. Fluorescent images were obtained using a 40×objective and a BZ-9000 fluorescence microscope and a 63×objective and a TCS-SP5 confocal microscope system (Leica, Wetzlar, Germany). For the analysis of infected cell ratios, the number of EGFP-positive cells was counted using a BZ-II Analyzer software (Keyence). For the analysis of neuronal transduction, the number of MAP2 and EGFP double-positive cells within the total number of EGFP-positive cells was counted manually. The primary antibodies (Abs) used were polyclonal rabbit anti-EGFP Ab (1∶1000; Life Technologies) and monoclonal mouse anti-microtubule-associated protein 2 (MAP2) Ab (1∶1000; Millipore, Bedford, MA, USA). The secondary Abs used were Alexa 488-conjugated goat anti-rabbit IgG (Life Technologies) and Alexa 555-conjugated goat anti-mouse IgG (Life Technologies) at 1∶500.

### Virus Administration to Embryonic and Post-embryonic Chicks

Fertilized eggs of Barred Plymouth Rock chicken (n = 55) were obtained from a local supplier (Shimizu Laboratory Supplies, Kyoto, Japan) and incubated in a humidified incubator at 37.5°C to desired stage [70]. Prior to viral injections, a small window was cut in the shell directly above the embryo. Viral solution (0.5–1.5 µl) containing 0.05% Fast Green (Nakarai tesque) was injected into the lumen of the neural tube near the ear vesicle using a sharp glass pipette attached to a Toohey Spritzer pressure system IIe (Toohey Company, Fairfield, NJ, USA). After injection, the window was closed with cellophane tape and embryos were incubated at 37.5°C. For the induced gene expression, 0.5 ml of Dox solution (0.1 mg/ml in PBS) was injected into the yolk sac using a syringe equipped with a 31-gauge needle. Embryos were harvested from eggs at specific stages. Whole brains were dissected and fixed with 4% paraformaldehyde in 0.1 M phosphate buffer for 3 days. The coronal sections including the middle regions of NL along the rostral-caudal axis were used for immunofluorescence studies.

For the striatum infection studies, we used White Leghorn chicks (Takeuchi Farm, Nara, Japan) at PHD5–6 (n = 12). For the IMM studies, we used Barred Plymouth Rock chicken (n = 3). Newly hatched chicks were used within several hrs after hatch. The chicks were deeply anesthetized with ketamine (15 mg/kg body weight, Daiichi Sankyo, Tokyo, Japan) and xylazine (7.5 mg/kg body weight, Wako Pure Chemicals), and fixed on stereotaxic apparatus. Stereotaxic coordinates for the striatal injection were as follows: 6 mm anterior from the bregma; 1.8 mm lateral from the midline; 5 mm ventral from the pial surface [71]. And for the IMM injection: 3 mm anterior from the bregma; 1 mm lateral from the midline, 2.5 mm ventral from the pial surface [71]. The viral solutions were injected over 5 min using Toohey Spritzer pressure system IIe.

### Immunofluorescence Study

After 24 hrs or 7 days from viral infection, chicks were perfused intraventricularly with 4% paraformaldehyde in 0.1 M phosphate buffer under deep anesthesia. Whole brains were dissected and post-fixed overnight. The fixed brains were cryoprotected with 30% sucrose in PBS overnight and embedded in O.T.C. compound (Sakura Finetek, Torrance, CA, USA). Frozen brains were cut into 40-µm-thick sections on a freezing microtome (CM1850; Leica) and processed as free-floating for immunofluorescence labeling as follows. The sections were permeabilized and blocked with PBS containing 10% normal goat serum and 0.3% Triton X-100 for 60 min at room temperature, and incubated with rabbit anti-EGFP (1∶1000; Life Technologies) and mouse anti-NeuN (1∶500; neuronal marker, Chemicon, Temecula, CA, USA) or rat anti-EGFP (1∶1000; Nakarai tesque) and rabbit anti-DsRed to visualize mCherry (1∶200; Clontech) in PBS containing 10% normal goat serum and 0.3% Triton X-100 overnight at 4°C. Following three washes with PBS, sections were incubated with Alexa Fluor 488-conjugated anti-rabbit IgG and 555-conjugated anti-mouse IgG or 488-conjugated anti-rat IgG and 555-conjugated anti-rabbit IgG (all 1∶250; Life Technologies) in PBS containing 0.5% normal goat serum and 0.1% Triton X-100 for 1 hr at room temperature. After three washes with PBS, sections were counterstained with DAPI, and mounted onto glass slides with Fluor Save mounting media (DAKO, Glostrup, Denmark). Images were obtained using 10×and 40×objectives with a BZ-9000 fluorescence microscope, and a 63×objective with a TCS-SP5 confocal microscope system. To quantify the EGFP-positive area per section, images through the section containing the injection site were tiled together using a BZ-II Analyzer. The tiled images were saved as 8-bit TIFF files (fluorescence intensity range 0–255) and the pixels (fluorescence intensity >99) were summed over the EGFP-positive area using NIH ImageJ software. The immunoreactive cells were counted manually.

### Statistical Analysis

Results were presented as mean ± SD. Statistical comparisons were performed with a two-tailed Student’s t-test. Results were considered to be statistically significant when p<0.05.

## Supporting Information

Table S1 **Raw data of** **Figure 1L** **.** Quantification of overall gene expression.
(DOC)

Table S2 **Raw data of** **Figure 1M** **.** Quantification of overall gene transduction rate.
(DOC)

Table S3 **Raw data of** **Figure 2B** **.** The neuronal transduction rates of A3V and LV are represented as the percentage of MAP2 and EGFP double-positive cells within EGFP-positive cells.
(DOC)

Table S4 **Raw data of** **Figure 3D** **.** Gene transduction after LV or A3V injection was quantified by measurements of EGFP-expressing area in the parasagittal sections containing injection sites.
(DOC)

Table S5 **Raw data of** **Figure 4F** **.** A3V transduction rates in the embryonic auditory nuclei were quantified as the percentage of EGFP-expressing cells within total NeuN-positive cells in each nucleus.
(DOC)
