# Supplementary material for: Avian Adeno-Associated Virus Vector Efficiently Transduces Neurons in the Embryonic and Post-Embryonic Chicken Brain
Source: PLoS One. 2012 Nov 7;7(11):e48730. doi: 10.1371/journal.pone.0048730 (PMC3492410; doi:10.1371/journal.pone.0048730)
Supplement: Table S1 — Raw data of Figure 1L . Quantification of overall gene expression. (DOC) [file pone.0048730.s001.doc]

**Table S1**

| Chicken | #1 | #2 | #3 | #4 | average | SD |
| --- | --- | --- | --- | --- | --- | --- |
| A3V | 59.5 | 56.2 | 56.0 | 61.2 | 58.2 | 2.6 |
| AAV2 | 0.0 | 0.0 | 0.0 | 0.0 | 0.0 | 0.0 |
| LV | 1.5 | 1.4 | 5.9 | 3.9 | 3.2 | 2.1 |

| ZebraFinch | #1 | #2 | #3 | #4 | average | SD |
| --- | --- | --- | --- | --- | --- | --- |
| A3V | 0.06 | 0.04 | 0.07 | 0.04 | 0.05 | 0.01 |
| AAV2 | 0.00 | 0.00 | 0.00 | 0.00 | 0.00 | 0.00 |
| LV | 0.02 | 0.03 | 0.04 | 0.02 | 0.03 | 0.01 |

| 293T cells | #1 | #2 | #3 | #4 | average | SD |
| --- | --- | --- | --- | --- | --- | --- |
| A3V | 0.0 | 0.0 | 0.0 | 0.0 | 0.0 | 0.0 |
| AAV2 | 0.2 | 0.3 | 0.1 | 0.2 | 0.2 | 0.1 |
| LV | 22.8 | 22.6 | 17.8 | 17.1 | 20.1 | 3.0 |
