# Supplementary material for: Avian Adeno-Associated Virus Vector Efficiently Transduces Neurons in the Embryonic and Post-Embryonic Chicken Brain
Source: PLoS One. 2012 Nov 7;7(11):e48730. doi: 10.1371/journal.pone.0048730 (PMC3492410; doi:10.1371/journal.pone.0048730)
Supplement: Table S2 — Raw data of Figure 1M . Quantification of overall gene transduction rate. (DOC) [file pone.0048730.s002.doc]

**Table S2**

| Chicken | #1 | #2 | #3 | #4 | average | SD |
| --- | --- | --- | --- | --- | --- | --- |
| A3V | 95.2 | 86.0 | 91.1 | 90.2 | 90.6 | 3.8 |
| AAV2 | 0.2 | 0.0 | 0.1 | 0.0 | 0.1 | 0.1 |
| LV | 24.6 | 28.5 | 28.9 | 19.6 | 25.4 | 4.3 |

| ZebraFinch | #1 | #2 | #3 | #4 | average | SD |
| --- | --- | --- | --- | --- | --- | --- |
| A3V | 5.9 | 10.9 | 10.0 | 13.1 | 10.0 | 3.0 |
| AAV2 | 0.8 | 0.6 | 0.3 | 0.3 | 0.5 | 0.2 |
| LV | 2.9 | 7.3 | 3.6 | 1.8 | 3.9 | 2.4 |

| 293T cells | #1 | #2 | #3 | #4 | average | SD |
| --- | --- | --- | --- | --- | --- | --- |
| A3V | 0.0 | 0.0 | 0.0 | 0.0 | 0.0 | 0.0 |
| AAV2 | 38.7 | 46.8 | 42.0 | 49.0 | 44.1 | 4.7 |
| LV | 21.7 | 28.7 | 25.9 | 30.8 | 26.8 | 3.9 |
