# Supplementary material for: Avian Adeno-Associated Virus Vector Efficiently Transduces Neurons in the Embryonic and Post-Embryonic Chicken Brain
Source: PLoS One. 2012 Nov 7;7(11):e48730. doi: 10.1371/journal.pone.0048730 (PMC3492410; doi:10.1371/journal.pone.0048730)
Supplement: Table S3 — Raw data of Figure 2B . The neuronal transduction rates of A3V and LV are represented as the percentage of MAP2 and EGFP double-positive cells within EGFP-positive cells. (DOC) [file pone.0048730.s003.doc]

**Table S3**

| Chicken | #1 | #2 | #3 | #4 | average | SD |
| --- | --- | --- | --- | --- | --- | --- |
| A3V | 96.6 | 93.2 | 88.6 | 86.0 | 91.1 | 4.7 |
| LV | 39.9 | 30.9 | 42.5 | 47.5 | 40.2 | 6.9 |
