# Supplementary material for: Avian Adeno-Associated Virus Vector Efficiently Transduces Neurons in the Embryonic and Post-Embryonic Chicken Brain
Source: PLoS One. 2012 Nov 7;7(11):e48730. doi: 10.1371/journal.pone.0048730 (PMC3492410; doi:10.1371/journal.pone.0048730)
Supplement: Table S4 — Raw data of Figure 3D . Gene transduction after LV or A3V injection was quantified by measurements of EGFP-expressing area in the parasagittal sections containing injection sites. (DOC) [file pone.0048730.s004.doc]

Table S4

| Striatum | #1 (hemisphere A) | #1 (hemisphere B) | #2 (hemisphere A) | #2 (hemisphere B) | average | SD |
| --- | --- | --- | --- | --- | --- | --- |
| A3V 5×10^7^ | 2.3 | 3.6 | 4.9 | 2.9 | 3.4 | 1.1 |
| A3V 5×10^8^ | 8.3 | 9.9 | 7.8 | 6.6 | 8.2 | 1.4 |
| A3V 5×10^9^ | 24.0 | 25.8 | 25.0 | 21.2 | 24.0 | 2.0 |
| LV 5×10^7^ | 0.1 | 0.1 | 0.2 | 0.0 | 0.1 | 0.1 |
