# Supplementary material for: Avian Adeno-Associated Virus Vector Efficiently Transduces Neurons in the Embryonic and Post-Embryonic Chicken Brain
Source: PLoS One. 2012 Nov 7;7(11):e48730. doi: 10.1371/journal.pone.0048730 (PMC3492410; doi:10.1371/journal.pone.0048730)
Supplement: Table S5 — Raw data of Figure 4F . A3V transduction rates in the embryonic auditory nuclei were quantified as the percentage of EGFP-expressing cells within total NeuN-positive cells in each nucleus. (DOC) [file pone.0048730.s005.doc]

**Table S5**

| NM | #1 | #2 | #3 | #4 | #5 | #6 | average | SD |
| --- | --- | --- | --- | --- | --- | --- | --- | --- |
| E1.5 | 0.0 | 0.0 | 4.0 | 0.0 | 0.5 | 1.1 | 0.9 | 1.6 |
| E2.5 | 82.4 | 92.7 | 82.9 | 76.1 | 89.0 | 88.0 | 85.2 | 5.9 |
| E3.0 | 62.0 | 84.3 | 60.6 | 47.2 | 68.5 | 72.9 | 65.9 | 12.5 |
| E3.5 | 49.6 | 52.4 | 61.0 | 55.6 | 65.3 | 71.3 | 59.2 | 8.2 |
| E4.5 | 19.1 | 23.8 | 16.4 | 2.0 | 6.5 | 6.9 | 12.4 | 8.5 |

| NL | #1 | #2 | #3 | #4 | #5 | #6 | average | SD |
| --- | --- | --- | --- | --- | --- | --- | --- | --- |
| E1.5 | 0.0 | 0.0 | 2.0 | 0.0 | 0.0 | 0.0 | 0.3 | 0.8 |
| E2.5 | 0.0 | 0.0 | 0.0 | 0.0 | 1.7 | 1.1 | 0.5 | 0.7 |
| E3.0 | 4.8 | 4.1 | 18.1 | 10.4 | 1.0 | 1.4 | 6.6 | 6.6 |
| E3.5 | 39.3 | 58.9 | 31.3 | 41.6 | 19.8 | 39.6 | 38.4 | 12.9 |
| E4.5 | 5.0 | 10.4 | 39.3 | 12.4 | 4.1 | 8.1 | 13.2 | 13.2 |

| NA | #1 | #2 | #3 | #4 | #5 | #6 | average | SD |
| --- | --- | --- | --- | --- | --- | --- | --- | --- |
| E1.5 | 0.0 | 0.0 | 0.3 | 0.0 | 0.0 | 0.0 | 0.1 | 0.1 |
| E2.5 | 10.9 | 16.5 | 18.8 | 12.2 | 23.9 | 9.4 | 15.3 | 5.5 |
| E3.0 | 44.1 | 30.4 | 20.3 | 9.9 | 39.1 | 39.9 | 30.6 | 13.2 |
| E3.5 | 55.4 | 39.9 | 80.6 | 72.3 | 63.3 | 56.8 | 61.4 | 14.2 |
| E4.5 | 67.8 | 72.5 | 60.1 | 26.9 | 37.2 | 31.9 | 49.4 | 19.7 |

| SON | #1 | #2 | #3 | #4 | #5 | #6 | average | SD |
| --- | --- | --- | --- | --- | --- | --- | --- | --- |
| E1.5 | 0.0 | 2.8 | 0.9 | 0.0 | 2.2 | 4.1 | 1.7 | 1.6 |
| E2.5 | 44.4 | 52.6 | 52.6 | 42.1 | 48.9 | 58.9 | 49.9 | 6.1 |
| E3.0 | 52.8 | 39.2 | 52.1 | 30.4 | 51.0 | 45.7 | 45.2 | 8.9 |
| E3.5 | 43.8 | 49.2 | 27.1 | 57.4 | 50.0 | 68.1 | 49.3 | 13.7 |
| E4.5 | 29.8 | 34.8 | 45.7 | 20.6 | 31.3 | 30.1 | 32.1 | 8.2 |
